# Teaching the true cost of medicines: white paper for a green and sustainable pharmacology curriculum

**DOI:** 10.3389/fphar.2026.1912700

**Published:** 2026-09-30

**Authors:** Ramamoorthy Rajkumar, Gavin S. Dawe

**Affiliations:** Department of Pharmacology, Yong Loo Lin School of Medicine, National University of Singapore, Singapore, Singapore

**Keywords:** curriculum, drug lifecycle, green, pharmacology, sustainable

## Abstract

The drug lifecycle imposes a growing environmental burden, evident in pharmaceutical pollution, escalating consumption of finite resources (energy, water, and raw materials) and a substantial carbon footprint. Meeting these challenges requires more than technological fixes; it demands a workforce trained to recognise, evaluate, and reduce the environmental impacts of medicines while safeguarding patient outcomes. However, recent scoping and systematic reviews consistently note a gap in health professions education: few curricula provide structured, evidence-informed approaches for integrating environmental sustainability and planetary health into pharmacology and related training. The discipline of pharmacology is uniquely suited for integrating green and sustainability principles because it sits at the decision-point where molecular mechanisms become real-world medicine use and environmental impact. This paper presents a white paper proposing a Green and Sustainable Pharmacology Curriculum and an actionable strategy for embedding sustainability principles within core pharmacology teaching. Targeted at biomedical scientists, pharmacists, and prescribers, the curriculum is designed to strengthen decision-making across the medicine lifecycle, including greener selection, rational use, and responsible disposal. We outline a curriculum development framework that applies Understanding by Design and outcome-based evaluation through four streamlined steps: (1) needs analysis, (2) curation of priority content topics, (3) constructive alignment of learning activities with intended competencies, and (4) assessment aligned to learning outcomes. By offering an adaptable blueprint for local implementation, this white paper aims to accelerate adoption by pharmacology departments worldwide. Ultimately, the curriculum seeks to shift learners from concerned global citizens to empowered pharmacologists with personal agency implement workplace changes that mitigate environmental harms and to serve as thought leaders advancing sustainable healthcare.

## 1 Introduction

Every medicine has a true cost invisible to patient and prescriber alike. Behind every therapeutic benefit lies an environmental ledger of pharmaceutical residues discharged into waterways, carbon emissions spanning global supply chains, and improperly discarded stockpiles leaching into ecosystems. The very molecules engineered to heal humankind and livestock are, across their lifecycle, quietly destabilising the ecological systems upon which human health ultimately depends. Yet the professionals who discover, produce, regulate, prescribe and dispense these medicines are, for the most part, trained without any systematic exposure to this paradox. Pharmacology curricula worldwide teach what drugs do to the body and what the body does to drugs but remain largely silent on what drugs do to the planet. Recent reports consistently identify this educational gap across pharmacy, nursing and medical curricula worldwide (Hashad et al., 2026; Egana et al., 2026; Niemi et al., 2025; Guilding et al., 2026), underscoring that upstream prevention through education, not downstream remediation alone, is the critical lever yet to be fully activated. This introduction first establishes conceptual clarity by delineating the terms *green*, *sustainable* and *ecological* as they apply to the drug lifecycle (Section 1.1), before examining the evidence compelling a curricular shift: the scale and severity of pharmaceutical pollution (Section 1.2), the emergence of ecopharmacovigilance as a paradigm for lifecycle stewardship (Section 1.3), the healthcare sector’s growing carbon footprint and its implications for climate change (Section 1.4), and the rationale for a pilot curriculum design framework (subsequent sections) that translates these imperatives into an actionable educational strategy.

### 1.1 Terminologies

The terms green, sustainable and ecological are frequently used interchangeably in pharmacology and pharmaceutical sciences literature. This conflation obscures important conceptual distinctions among these terms. The term *green* in the pharmaceutical context derives from the foundational concept of green chemistry defined by the International Union of Pure and Applied Chemistry (IUPAC) as “the invention, design, and application of chemical products and processes to reduce or to eliminate the use and generation of hazardous substances.” Aligning to the 12 principles of green chemistry (Anastas and Warner, 2000), green in the context of drug lifecycle may be defined as the design, synthesis, manufacture and formulation of pharmaceutical products and processes that minimise or eliminate the use and generation of hazardous substances, reduce waste, conserve energy and lower unintentional toxicological risk. The focus is on the intrinsic chemical and process-level properties of the pharmaceutical entity itself, from molecular design through to inherent biodegradability and it extends to disposal and environmental fate insofar as molecular design determines degradability and ecotoxicity. Sustainable in the context of the drug lifecycle pertains to pharmaceutical practices, systems and policies that integrate environmental stewardship, social equity and economic viability across the entire drug lifecycle (from discovery through environmental fate), such that present therapeutic needs are met without compromising the health, resources, or opportunities of future generations (Keeble, 2007). Ecological, in the context of the drug life cycle, encompasses the interactions between pharmaceutical substances, including active pharmaceutical ingredients, their metabolites, excipients and degradation products and the living organisms and abiotic processes within ecosystems. It encompasses ecotoxicological effects on non-target species, bioaccumulation, persistence and disruption of ecosystem functions resulting from pharmaceutical release into the environment. Sustainable subsumes green (chemical, process and design dimensions) but extends beyond it to include systemic (environmental, socioeconomic and ethical dimensions), while ecological focuses on organism-environment interactions with pharmaceuticals.

### 1.2 Pharmaceutical pollution

The healthcare sector is no exception to various other global sectors that strive to achieve eco-friendly (green) processes that do not adversely impact the resources of future generations (sustainability). Pharmaceuticals (active pharmaceutical ingredients, excipients, solvents and related compounds) that are released at various stages of their lifecycle, namely, drug discovery, manufacturing, preclinical and clinical development and medicinal use and disposal, accumulate in and pollute the environment (Orive et al., 2022; Silva et al., 2012; Wilkinson et al., 2022; Tyumina et al., 2023; Wang et al., 2024; Kümmerer, 2010; Egana et al., 2026; Amin and Rahman, 2026). Veterinary pharmaceuticals and biological medicinal products are no exception because of their role as environmental pollutants (Oaks et al., 2004; Kuhler et al., 2009; Plaza et al., 2022). At least 34 pharmaceuticals and potential endocrine-disrupting compounds were detected in the source, finished or tap water samples of 19 US drinking water treatment plants (Benotti and Snyder, 2009; Benotti et al., 2009). Pharmaceuticals in the environment are a global issue, with a total of 631 substances detected in 71 countries, among which diclofenac is the most common pharmaceutical (in 50 countries) and carbamazepine, ibuprofen, sulfamethoxazole and naproxen are found in at least 45 countries (Aus Der Beek et al., 2016). There is conclusive evidence that pharmaceuticals and related products released into the environment reach living organisms (Silva et al., 2017; Blanco et al., 2023), in which they are known to cause behavioural changes (Kellner et al., 2015) and various organ- and gene-related toxicities (Liao et al., 2023; Domingo-Echaburu et al., 2021). Health promotion to reduce pharmaceutical use, ecodirected sustainable prescribing if drug use is deemed necessary, the selection and development of greener and sustainable medicines, the interception of pharmaceutical residues before they reach water sources, targeted (chemical-specific) wastewater management with low-cost natural sorbent materials and changes in policies to speed up implementation were listed as strategies to address the root causes of poor health and reduce pharmaceutical use (Helwig et al., 2024). Two decades of monitoring across 91 estuaries in 26 countries confirm that the very drugs designed to sustain human health are now accumulating in the ecosystems we depend upon, with hundreds of pharmaceuticals contaminating estuarine waters at levels that pose high ecological risk and correlate strikingly with regional poverty (Adedipe et al., 2024). Alarmingly, the footprint of pharmaceutical pollution extends beyond therapeutic agents to encompass drugs of abuse, with stimulants and opioids now detected across hundreds of rivers worldwide (Niroula et al., 2025). Although compound-specific interactions, mixture effects, long-term exposures, and ecosystem-level consequences remain critical knowledge gaps, the ecotoxicological impacts of pharmaceutical contamination and its role in propagating antibiotic resistance are increasingly supported by empirical evidence (Gaw et al., 2014). Global surface waters are now permeated by a complex cocktail of pharmaceuticals, illicit drugs and persistent synthetic chemicals at concentrations that pose demonstrable ecological risk, underscoring the sheer scale and urgency of pharmaceutical pollution as an environmental crisis (Monk et al., 2025). While downstream efforts, such as refining the methods of pharmaceutical detection, identifying the affected ecologies, estimating the actual levels of contamination and determining the magnitude of adverse impacts of low concentrations of chemical cocktails, are still in progress, collective action to reduce pharmaceutical pollution, an upstream effort, is inevitable. It has been argued that increasing pharmaceutical pollution directly and indirectly influences many (12 out of 17) of the sustainable development goals set by the United Nations under the 2030 Agenda for Sustainable Development (Domingo-Echaburu et al., 2021). Taken together, the evidence compels an inescapable conclusion: the scale, ubiquity and ecological severity of pharmaceutical pollution demand a shift from reactive remediation to proactive vigilance, one that systematically monitors and mitigates the environmental impact of drugs throughout their entire lifecycle.

### 1.3 Ecopharmacovigilance

Recognition of the potential negative impact of active pharmaceutical ingredients (APIs) on the environment has led to the emergence of the term ecopharmacovigilance (Garcia et al., 2024; Jena et al., 2019; Holm et al., 2013; Kuhler et al., 2009; Daughton and Ruhoy, 2008). Historically, this surge of awareness was triggered when diclofenac residues were linked to the calamitous decline of three species in the vulture population, driving them to the endangered species list. The residues reach them through the drug-treated livestock on which the vultures scavenge (Oaks et al., 2004). The preclinical phase includes the application of principles of green chemistry in medicinal chemistry (Bryan et al., 2013), the use of multiple cheminformatics methods in drug discovery (Lubick, 2008; Aliagas et al., 2017) and iterations of processes in pharmaceutical manufacturing (Bengtsson-Palme et al., 2018). In the clinical phase, it includes implementing ecopharmacovigilance measures (Holm et al., 2013); assessing, incentivising (to reduce), reviewing and monitoring the carbon footprints of clinical trials (Adshead et al., 2021); monitoring and refining prescribing practices for the rational use of medicines (Daughton and Ruhoy, 2011; Jain et al., 2015; Fisk et al., 2022); and collaborative efforts in the healthcare industry as a whole (Daughton and Ruhoy, 2008; Lertxundi et al., 2021), including the pharmaceutical waste management system (Sapkota and Pariatamby, 2023). To it cap it all, a need for holistic, coherent policies and regulatory frameworks (Jose et al., 2020; Miettinen and Khan, 2021; Malmqvist et al., 2023) has also been proposed. Currently, there is a pressing need to implement active ecopharmacovigilance strategies to minimise the release of pharmaceuticals from various pharmacological classes (Silva et al., 2012; Wang et al., 2017; Wang et al., 2021; Wang et al., 2018; Wang et al., 2019). Reducing environmental risk presents one of the main challenges in drug safety and evaluation in the future, and ecopharmacovigilance has the potential to guide more holistic sustainable decision-making (Garcia et al., 2024). These observations drive us toward the idea of equipping healthcare professionals with a holistic curriculum that integrates green and sustainability principles.

### 1.4 Carbon footprint and climate change

In addition to environmental pollution due to increasing medical waste, many practices in healthcare systems directly and indirectly contribute to the carbon footprint (Rodriguez-Jimenez et al., 2023; Carrasco-Amador et al., 2022; Keil et al., 2024; The Lancet Digital Health, 2023). A few recently published examples of processes in the healthcare sector that significantly contribute to the carbon footprint include surgical operations (Rizan et al., 2020; Savio et al., 2026) anaesthesia and critical care (McGain et al., 2020), radiology (Woolen et al., 2023) and gastrointestinal endoscopy (Park and Cha, 2023). To reduce the environmental impact of the healthcare sector to acceptable levels, a larger-scale transformation from curative to preventative healthcare services is necessary, and the role of AI in such a transformation has yet to be explored (Bratan et al., 2024). The European Society of Intensive Care Medicine (ESICM) presented a green paper that presents a structural approach for intensive care units to reduce their environmental impact to achieve high-quality patient care with an emphasis on energy efficiency and sustainable procurement and waste reduction (De Waele et al., 2024). Among the various proposed strategies for the upstream prevention of negative impacts and for addressing sustainability challenges, the inclusion of the ecological footprint, climate change and sustainable healthcare as core components of medical education (Dunne et al., 2023; Lunghi et al., 2025; Sarcone et al., 2025) has drawn our interest. Integrating green and sustainable principles into undergraduate medical education would cultivate awareness of these emerging concerns and equip medical and healthcare professionals with strategies to address underlying issues, thereby providing holistic service to society and the environment.

## 2 Literature search

A literature search in PubMed and ScienceDirect with the key words “sustainability OR sustainable practices AND green AND pharmacology” was performed alone or in combination with one or more keywords from the following list: “pharmacovigilance, education, curriculum, programme, design, evaluation, models, (pedagogy OR pedagogical), strategy, (needs analysis OR needs assessment analysis), constructive alignment, interdisciplinary, medical education, environment, pollution, carbon footprint, drug lifecycle, core concepts, understanding by design, (prescription OR prescribing practices), (dispensing OR dispensing practices) and planetary health.” The objectives of the literature search were (1) to find publications that evaluated the role of education and the curriculum in managing the challenges of pharmaceutical pollution and carbon emissions and the emerging role of ecopharmacovigilance; (2) to examine the awareness and extent of incorporation of green and sustainability principles in the pharmacology curricula of undergraduate medical and life science education; and (3) to review existing curriculum designs, their integral elements and evaluation strategies to identify a plausible recipe for a framework that is suitable for incorporating green and sustainability principles in medical education. The papers were read, and cross-references to other publications pertaining to the theme of this review were followed.

## 3 Pilot curriculum design framework: white paper

A pilot curriculum design framework was developed using appropriate models and a succinct sequence of steps, including needs analysis, curation of content topics, constructive alignment of activities, assessment and learning outcomes, and curriculum evaluation. This white paper is intended to inspire pharmacology departments worldwide to adopt this strategy to develop a green and sustainable pharmacology curriculum to complement medical, dental, pharmacy and nursing education.

### 3.1 Need for a green and sustainable pharmacology curriculum

The literature search identified publications that examined or reviewed awareness or need for green and sustainability principles among medical and healthcare professionals and reported that a few universities incorporated them in postgraduate curricula. In a questionnaire-based study involving two scenarios with fictitious medicines as options for the treatment or symptom relief of minor ailments, compared with medical and healthcare students, pharmacy students gave higher scores for environmentally harmful medicine (Hakonsen and Hedenrud, 2024). Furthermore, a cross-sectional study in five pharmacy schools that assessed students’ knowledge of drug pollution revealed that although students considered drug pollution an important issue and expressed interest in acquiring knowledge, their awareness of key concepts was poor (Abajo et al., 2024). These trends, although localised, necessitate an interdisciplinary curriculum to help pharmacy professionals make eco-consciously informed decisions. A 20-item questionnaire to determine awareness of ecopharmacovigilance and ways of storing and disposing of unused and expired drugs revealed that Croatian students had relatively better knowledge of these practices than students in other similar studies did. Despite the small sample size of 80 students (medicine, dental and pharmacy), studies have revealed the need to implement ecopharmacovigilance in healthcare and medical students’ curricula and strengthen their foundational knowledge (Durdov et al., 2024). Ladhar et al. reported the zeal expressed by students of the Doctor of Pharmacy programme on the topic of the environmental impact of medications, to which students had limited exposure in the programme, underscoring a gap in the curriculum (Ladhar et al., 2025). A cross-sectional study in Swedish universities revealed that although education on the environmental impacts of pharmaceuticals was evident to an extent, there is room to further develop and integrate environmental themes ideally, using an interprofessional, lifecycle perspective, to better equip future professionals to address these challenges (Jazzaa et al., 2025).

Recent reports have consistently identified a clear gap in current training and have urged the explicit integration of environmental impact (Villen et al., 2025) or sustainability principles into pharmacy (Hashad et al., 2026; Rajiah and McArthur, 2026) and nursing (Akore Yeboah et al., 2024) and into all healthcare-related curricula (Egana et al., 2026; Niemi et al., 2025) to better prepare graduates for environmentally responsible, future-ready practices. In addition to raising awareness and improving the infrastructure for medication disposal, the inclusion of safe medication disposal practices in academic curricula is crucial for reducing the environmental and public health risks of improper medication disposal (Hiew and Low, 2025). In accordance with the professional knowledge provided by the General Medical Council (United Kingdom), newly qualified doctors must be able to apply the principles, methods and knowledge of population health and improve health and sustainable healthcare to medical practice (General Medical Council, 2018). Sustainable changes to reduce the carbon footprint may be achieved in prescribing practices, regulatory meetings and in the next-generation of environmentally conscious healthcare professionals. Notably, the teaching sector was recognised as one of the targets for sustainable change among other components of healthcare, such as hospitals, pharmacies and regulatory bodies, to facilitate awareness among the next-generation of healthcare professionals of the environmental impact of drugs (Adeyeye et al., 2022). There is a clear gap and a timely opportunity for universities to more explicitly define and strengthen their role in addressing sustainability within curriculum units, ensuring that sustainability is embedded across the full spectrum of pharmaceutical activities (Almeida, 2026).

Some universities have incorporated environmental sustainability into their curricula. The University of Huddersfield, Department of Pharmacy, integrated environmental sustainability into its master’s programme by viewing topics through an environmental lens instead of introducing new content, which was achieved by (1) promoting collaboration with geographers, chemists, economists, computer scientists, nurses, medical staff and others for interprofessional student work; (2) involving student and patient representatives on topics that explicitly geared towards environmental research projects involving students and patients; and (3) using existing topics within the curriculum that are applicable to climate change. Similarly, the University of Helsinki successfully integrated green aspects into its higher education in pharmaceutical and medical sciences. They achieved the formation of a task force that steered societal interaction and promoted new research collaboration; reformed the curriculum through a previously established framework; and promoted the long-term and continuous engagement of faculty leaders through a cohort of students, faculty and stakeholders. Finally, the prospective changes were disseminated as positive outcomes as collective expertise for the profession. Suggestions for the adaptation of such a concept in other curricula include the need for constant, long-term support and motivation by peer-teachers and a task force for a change in mindset; valuing students’ needs and interests; engaging them early in planning and implementing curriculum reform; and finally justifying teaching methods and linking them to learning outcomes such that cognitive objectives and generic skills are met (Sivén et al., 2020; Lapatto-Reiniluoto et al., 2022). Apart from the aforementioned universities, which directly integrated climate and health topics into the curriculum, other universities introduced these topics in mandatory course work, *ad hoc* lectures, elective courses (with lectures, self-study, video, reflection and field visits), case studies or extracurricular activities (Dunne et al., 2023; Mathers et al., 2023). To include it as core course material or as continuing education activities, green and sustainability principles need to be identified in professional, educational and accreditation standards (Mathers et al., 2023). The hybridisation of green and sustainable aspects in to the pharmacology curriculum must not be perceived as a burden because of the expanding content but rather as an opportunity to embrace innovative strategies to create awareness of and equip professionals to address this emerging issue (Fasinu and Wilborn, 2024). However, there was a clear lack of publications addressing the later stages of curriculum development, with only two articles describing a complete curriculum development cycle. A systematic review focusing on the educational approaches applied to sustainable healthcare education within health profession curricula concluded that despite the range of approaches used to deliver sustainable healthcare education, no superior approach was identified (Bray et al., 2023). Integrating green chemistry principles into pharmaceutical education equips future professionals with the ability to design safer, less toxic drugs; adopt cleaner and more resource-efficient manufacturing processes; and make ethically informed decisions that balance therapeutic efficacy with environmental responsibility (Guilding et al., 2026).

While the literature reflects a growing interest in embedding green and sustainability principles within medical and life science education, a comprehensive and interdisciplinary curriculum that systematically integrates these principles into medical training remains conspicuously absent. Pharmacology, a discipline that is widely recognised as the bridge between basic sciences and clinical medicine, presents a uniquely strategic entry point for this integration. We propose hybridising the pharmacology curriculum with green process and sustainability principles, contending that this is not merely an enhancement but a pedagogical imperative: future biomedical scientists and healthcare professionals must be equipped with a critical awareness of the environmental consequences of the drug lifecycle and empowered to make ecologically informed decisions at every stage, from drug design and manufacturing to prescribing, consumption and disposal. With regulatory frameworks, technological innovations and industry-led initiatives alone proving insufficient to fully mitigate the environmental footprint of pharmaceuticals, embedding sustainability directly into the education of those who will design, produce and prescribe these drugs remains the critical lever yet to be fully activated.

### 3.2 The foundational normative question: Why should pharmacologists and healthcare professionals care?

Before learners engage in the technical dimensions of green and sustainable pharmacology (e.g. life-cycle assessment, pharmaceutical pollution pathways, or sustainable prescribing strategies) they must first confront a foundational normative question: why should pharmacologists and healthcare professionals care about the environmental consequences of medicines?

This question is the ethical anchor upon which the entire curriculum rests. Amid rising disease burdens, shifting population needs, evolving treatment modalities and persistent staff shortages and resource constraints, healthcare professionals already overwhelmed by the delivering quality patient care, may perceive the ecological crisis and its gradual manifestations as beyond their scope of responsibility (Born et al., 2024; The Lancet Global, 2023; Agyeman-Manu et al., 2023). Gaining insight into how health professions students perceive planetary health and sustainability is a necessary first step towards meaningful curricular integration to manage the triple planetary crisis namely climate change, pollution and biodiversity loss and it is recognised that missing this crucial educational shift would leave the healthcare sector inadequately positioned to address the escalating ecological crises (Ihsan et al., 2024). Further, the longstanding principle of “first, do no harm” must be reinterpreted to encompass the environmental harms generated by healthcare systems themselves (Thornber et al., 2022). Given that protecting natural systems is a prerequisite for sustaining human health, the proposed curriculum necessarily engages with value judgments about professional responsibility, intergenerational justice, ecological stewardship and the evolving goals of medicine in the Anthropocene (Whitmee et al., 2015).

Pharmaceutical pollution raises profound questions of moral responsibility for prescribers and the pharmaceutical industry alike (Malmqvist et al., 2023), while distinct professional duties now extend across the full product lifecycle—from producers and regulators to prescribers (Parker and Miller 2024; Piët et al., 2024). Embedding environmental and planetary health education within health professions training is therefore increasingly framed not as optional enrichment but as a social imperative and core professional competency (Prifti et al., 2025; Naidu and Ramani, 2024).

We therefore recommend that the curriculum begins with an introductory ethics and professional responsibility module that invites learners to critically examine questions such as: Why should environmental harm be considered part of the “true cost” of medicines? Does the professional duty to benefit patients extend to safeguarding future patients and the ecosystems upon which health depends? How should pharmacologists navigate tensions between immediate patient-centred outcomes and longer-term ecological harms? These normative foundations have been advocated as essential starting points for embedding sustainability into pharmacy and pharmacology education specifically (Mathers et al., 2023; Dunne et al., 2023; Almeida, 2026), and for health professions training more broadly (Mulu and Kivuva, 2025). By positioning these questions as the curriculum’s starting point, learners develop personal conviction and professional identity around sustainability before acquiring technical competencies. This sequencing can such as strengthen intrinsic motivation, deepen engagement and foster the sense of personal agency that transforms passive recipients of sustainability content into reflective professionals who understand why change is necessary and feel professionally compelled to enact it.

### 3.3 Curriculum plan

#### 3.3.1 Needs analysis

The first and foremost or common element among the various curriculum models is needs analysis. Student-centric analysis can be performed with surveys of current students (undergraduate health professions and life sciences and graduate students), potential students and potential employers of students (local and regional healthcare systems, the pharmaceutical industry, regulatory authorities, etc.) on their overall expectations of the green and sustainable pharmacology course and what should be covered in a green and sustainable pharmacology curriculum. The needs analysis will involve questionnaire-based surveys and/or personal interviews (Cargonja et al., 2021; Chan et al., 2022; Civaner et al., 2022; Pepper, 2021; Scott et al., 2019; Valois et al., 2016). The items of the questionnaire will essentially include (i) a brief description of the course that is intended to be launched, (ii) open-ended questions on stakeholder expectations from a green and sustainable pharmacology course on the basis of their perspective, (iii) expectations of the skills and knowledge the students need to acquire (expected learning outcomes) to play an appropriate role in the organisation, (iv) specific elements of the curriculum that would help achieve the learning outcomes and (v) an item to rank the list of proposed content topics (see below) and options to propose new topics. The outcome of a qualitative analysis of needs assessment forms a sound basis for identifying the elements and designing the curriculum.

#### 3.3.2 Interdisciplinary team

Given the interdisciplinary nature of the subject, pharmacologists must partner with faculty teams bringing expertise in green chemistry, lifecycle assessment, sustainable design and manufacturing, environmental toxicology, bioethics, and clinical practice, alongside policymakers and regulators responsible for waste discharge, resource management, and environmental quality standards (Figure 1). The interdisciplinary team offers students (1) broad theoretical coverage of the drug lifecycle; (2) opportunities to develop design and systems thinking; (3) interdisciplinary research experience that mirrors coordinated workplace practice; and (4) ethical guidance that moves them from identifying what is greener to reasoning rigorously about what ought to be done when environmental and patient-centred goals conflict. A questionnaire survey of directors of interdisciplinary programmes revealed that (1) students are more sensitive to ethical issues; (2) they have the ability to synthesise or integrate concepts; (3) they have enlarged perspectives and greater awareness of public issues; (4) they have more creative, original or unconventional thinking; (5) they have greater humility or listening skills since they become conscious of the partial validity of any perspective and how it can be enriched by learning with others; and (6) they are more sensitive to bias (Newell, 1990). Notably, curriculum needs analysis aimed at addressing an emerging concern that focuses on the transformation of students’ mindsets and the adoption of a consultative (student and faculty feedback) approach for curriculum evaluation will render dynamicity to team composition. A core team of educators with diverse expertise will be the driving force for designing, implementing and iterating the curriculum to meet the overall objectives of the course.

**FIGURE 1:**
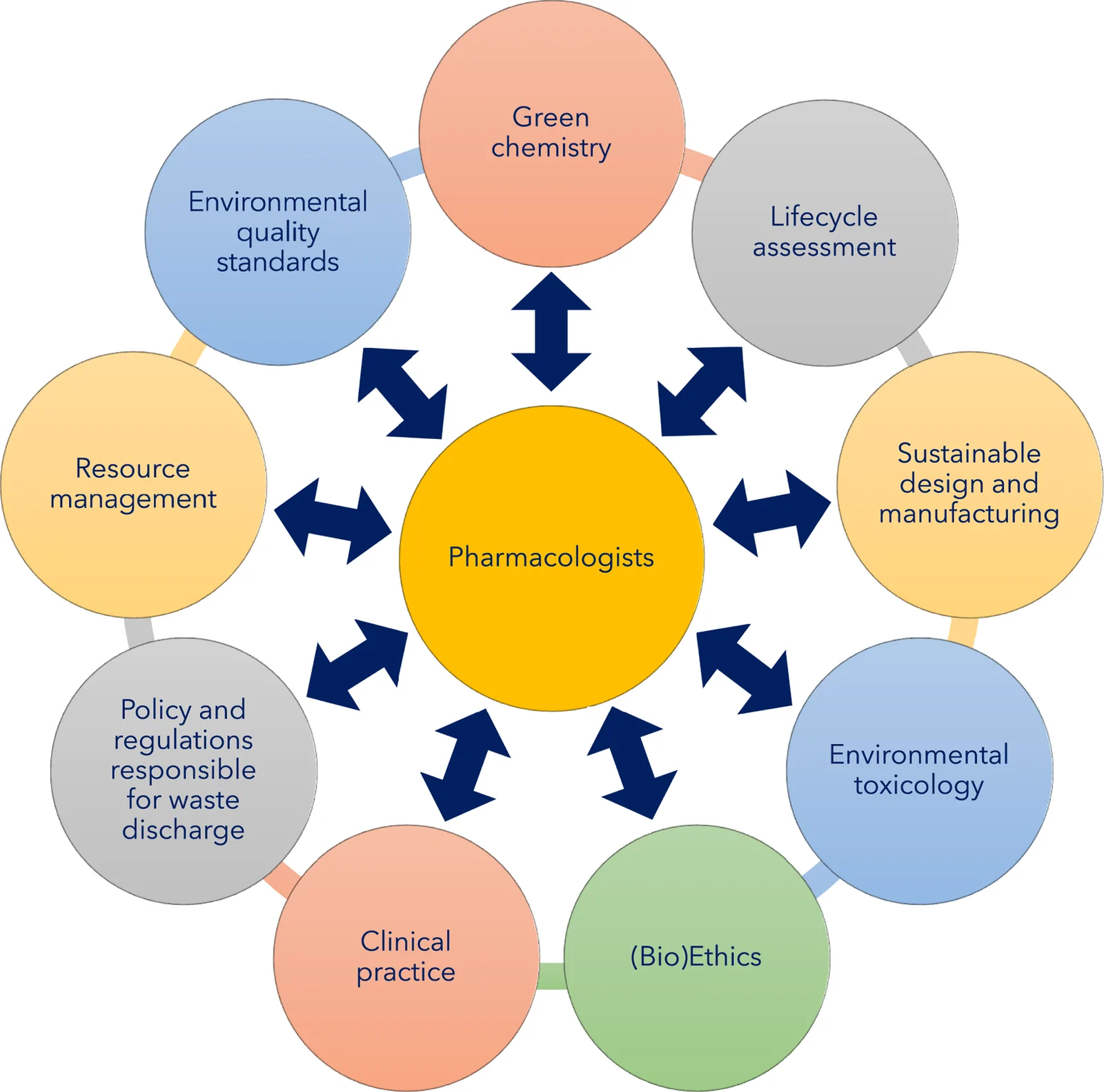
Interdisciplinary team: Expertise to partner with pharmacologists to form the proposed interdisciplinary team for the green and sustainable pharmacology curriculum.

#### 3.3.3 Design

There are several considerations in selecting a curriculum design model from a plethora of available models (Kelting-Gibson, 2013; Andrews and Goodson, 1980). Models may be classified as prescriptive or descriptive (Prideaux, 2003), linear (Tyler, Dick and Carey, and analysis, design, development, implementation and evaluation [ADDIE] models), mutually interactive (Taba’s model), nonlinear (Kemp model), value driven (John Goodlad’s model), cyclic (Wheeler’s model) (Wiggins and McTighe, 2005), or student-centric with interrelated components (Kerr’s and ADDIE models). Every model of curriculum design has its own (dis)advantages, and models do have overlapping features. With no direct body of evidence that supports one best choice for framing a curriculum, the focus should be on developing one that is appropriate for the purpose (Andrews and Goodson, 1980) and context (Grant, 2018; Edmonds et al., 1994). We propose the use of the understanding by design (UbD) model, which has three stages: (1) identifying the desired results, (2) determining acceptable evidence and (3) planning learning experiences and instructions (Wiggins and McTighe, 2005). This backward design model is appropriate for the green and sustainable pharmacology curriculum over other models for the following reasons.

The UbD model (1) is relatively easy for beginners to adopt in instructional design with fewer steps and complexity than the Dick and Carey (Dick, 1996), Kerr’s or Kemp models are; (2) focuses more on depth of understanding and adaptability in learning, which is more relevant to higher-order learning outcomes than the behavioural focus of the Dick and Carey model (Dick, 1996; Dick, 2013) or predefined objectives and measurable outcomes offered by Tyler’s model (Tyler, 2013); (3) has an outcome-focused backward design that aligns well with the outcome-based education (OBE) approach widely adopted as a framework for evaluating medical education curricula (Iqbal et al., 2020; Harden, 2007; Harden, 2009), facilitating appropriate qualitative and quantitative evaluations (Japee and Oza, 2021) of green and sustainable pharmacology curriculum outcomes. The backward design process is an advantage over the linear or inductive approach (of Tyler’s or Dick and Carey’s models) or the cyclical or dynamic process, which focuses on interactions between the components but may not start with outcomes (Wheeler’s, Kemp’s and Kerr’s models); (4) is less prescriptive but more flexible for adaptation to different curricula, especially those that are interdisciplinary than rigid models (Tyler’s) that focus on specific objectives and outcomes; (5) compared with Kerr’s model, it is explicitly centred on student engagement, which promotes active learning by engaging learners through essential questions and real-world applications; (6) integrates assessment intrinsically into the design process (focusing on evidence of understanding before planning instruction), which is different from Tyler’s and Wheeler’s models; (7) shares the flexibility and builds on existing knowledge aspects of the constructivist approach (Kaufman, 2018) but is unique because of its backward design, which starts by identifying the desired results that are more appropriate for a green and sustainable curriculum; (8) is relatively new (for at least a couple of decades) and its resources are still being actively developed; hence, it is more likely to permit iterations to the resources on the basis of observations from developed curricula that address emerging issues.

The UbD framework has been successfully applied and well received across many universities. Some examples of science and medical courses include a nonmajor biology course at Columbia College, Chicago (Minbiole, 2016); a health education course at Saint Leo University (Crossman, 2016); a master’s degree in a nursing education programme at Kennesaw State University, Georgia, United States (White and Maguire, 2021); a recent interdisciplinary integration of basic sciences with therapeutic content at Ohio State University (Cline et al., 2023); and multiple programmes and courses at the Baylor College of Medicine (Newell et al., 2024).

#### 3.3.4 Proposed content list

The earlier sections provide evidence that green and sustainability issues in the field of medicine are matters of emerging concern. The goal of generating eco-conscious healthcare professionals and biomedical scientists to achieve upstream prevention of the issue, rather than downstream mitigation of pharmaceutical pollution, simultaneously strikes a balance between the ecological footprint and maximal patient benefit but requires coordinated effort from all the actors involved in the drug lifecycle. Education on this emerging issue provided early in the career prepares professionals to face the ground reality with transformed mindsets (discussed below). In addition to achieving symbiosis with health services and communities and providing values that enhance the provision of health services, the curriculum must be responsive to changing values and expectations in education (Prideaux, 2003). The current expectation of hybridisation of the pharmacology curriculum with sustainability aspects places a significant demand on medical educators, especially clinical pharmacologists (Bakkum et al., 2022). The selection of a single model of curriculum design for interdisciplinary medical education from a plethora of studies (Kelting-Gibson, 2013; Prideaux, 2003) is crucial. In addition, the list of considerations in curriculum design is continuously expanding. For example, while design thinking involves starting from practical situations and problems, exploring the potential needs and challenges of users and valuing human-centred design and innovative solutions, the inclusion of design-based learning (a combination of design thinking and design practice) as the core of interdisciplinary curriculum design was proposed as a concrete, workable and essential pedagogical strategy in the future (Wang, 2024). A comprehensive and interdisciplinary curriculum must provide the following.

1. Foundational topics in the fields of sustainability, green principles and pharmacology should be considered to identify and understand the impact of this emerging concern.
2. Insight into the drug lifecycle and opportunities for applying green and sustainable principles. In short, students should be guided to observe the drug lifecycle through green and sustainable lenses.
3. A complete account of the available methods for detecting pharmaceutical pollution is currently needed to identify sustainability challenges in the drug lifecycle and strategies currently used to mitigate them.
4. The basis for innovation is to develop new and/or refine existing strategies in the areas of detection, implementation, monitoring, ethical considerations and regulatory matters/policy making with respect to all stages of the drug lifecycle.
5. Both theoretical knowledge and a peek into practical applications (via experiential learning) enable students to spearhead sustainability roles in industry, academia, clinics and regulatory bodies.

#### 3.3.5 Constructive alignment

The curriculum needs analysis phase of curriculum development identifies expected learning outcomes that reflect the expectations put forth by stakeholders (industry partners, hospitals and regulatory agencies). Achieving broad learning outcomes, such as changes in the mindset or attitudes of students to wear green and sustainable lenses to make a change in their workplace, has an antecedent step, namely, constructive alignment (Biggs, 1996; Biggs, 2012). This outcome-based approach has two main principles: (1) knowledge is constructed by students through their own learning activities but not merely transmitted by the teacher; and (2) the intended outcomes of teaching must be stated upfront, and teaching methods and assessments need to be aligned with the outcome required to be met (Biggs and Tang, 2015). As noted for green analytical chemistry, a rigorous effort from educators is needed to develop curricula that allow students to visualise how green thinking impacts different facets of the discipline, thereby increasing the likelihood of shaping professionals who can resolve emerging issues (Dicks, 2023). This approach is known to be beneficial in large-scale and/or foundational fields such as microbiology (Wang J. T. et al., 2013), anatomy (Biggs and Tang, 2015), family medicine (Gilic et al., 2022) and design thinking in medical curricula (Squalli Houssaini et al., 2024). Students in constructively aligned courses are more likely to adopt deep learning approaches (Wang X. et al., 2013). Following this method, we attempted to (1) define the intended learning outcomes (ILOs) of the green and sustainable pharmacology course (see Supplementary Table S1) and align them with (2) teaching or learning activities likely to lead to the ILOs and (3) assessments of the students’ actual learning outcomes to determine how they matched the learning intended, as depicted in Figure 2.

**FIGURE 2:**
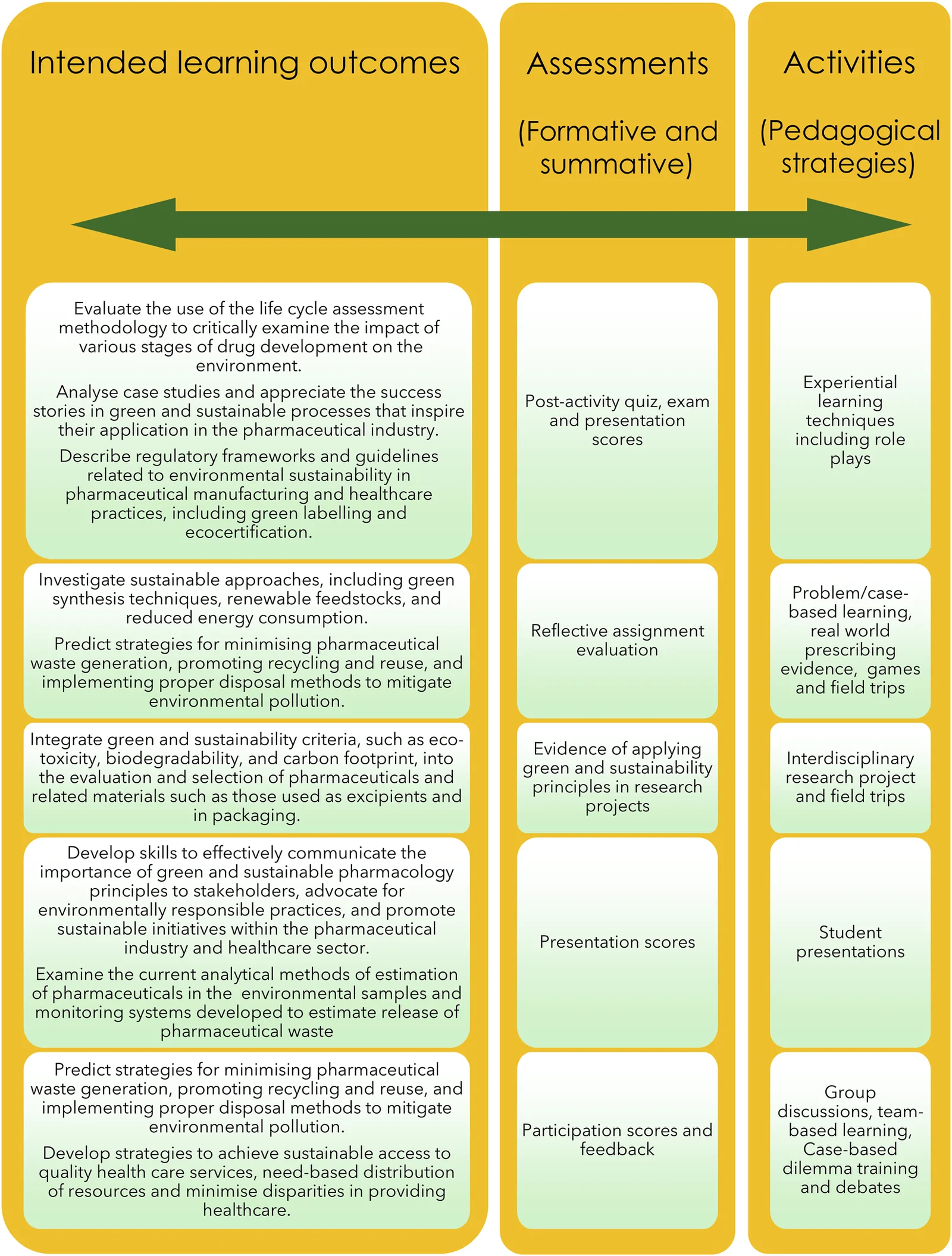
A suggested constructive alignment plan for green and sustainable pharmacology course design.

#### 3.3.6 Curriculum evaluation

The evaluation of the effectiveness of a curriculum is a systematic process. One suitable method for curriculum design would be to adopt an eclectic, outcome-focused strategy that leverages the strengths of multiple established models (Glatthorn et al., 2015). In our proposed implementation, we draw primarily from the UbD model (Wiggins and McTighe, 2005), which emphasises designing curricula with the desired learning outcomes as the starting point. UbD focuses on “backward design,” where educators first identify the goals and outcomes, then determine the evidence required to demonstrate the achievement of these goals and finally design instructional activities to achieve them. To enhance the UbD model’s focus on outcomes, elements from the systematic curriculum design model (Dick, 2013) are incorporated. This model emphasises a detailed and structured approach to curriculum development, including ADDIE. Its systematic nature provides a rigorous framework for evaluating the alignment of learning outcomes with instructional strategies and assessment tools. Additionally, principles from the OBE model (Iqbal et al., 2020; Japee and Oza, 2021) are integrated to ensure that all components of the curriculum (content, pedagogy and assessment) are explicitly aligned with the intended learning outcomes. OBE focuses on achieving clear, measurable outcomes that reflect what learners are expected to know, do and value at the end of a learning process. By combining these models, the proposed approach seeks to balance the conceptual clarity and outcome focus of UbD, the methodological rigour of the systematic design model and the accountability and alignment emphasised in OBE. This eclectic strategy allows for a more comprehensive and adaptable curriculum design that can cater to diverse educational settings and objectives. Ideally, the data collection and analysis could use a mixed methods approach (Creswell and Guetterman, 2019; Tashakkori and Teddlie, 1998), specifically a convergent design (Creswell and Guetterman, 2019), which involves collecting and evaluating qualitative and quantitative data concurrently or in parallel and merging the results for comparison and interpretation of convergence and divergence. The sources of evidence for the evaluation of the developed curriculum (Glatthorn et al., 2015; Iqbal et al., 2020; Japee and Oza, 2021) are summarised in Table 1.

**TABLE 1:** Sources of evidence for curriculum evaluation.

Source of evidence	Methods
An effectiveness indicators checklist	Bradley’s Effectiveness Model for curriculum evaluation (Glatthorn et al., 2015) provides ten key indicators that measure the effectivenes of a developed curriculum. Meanwhile, the OBE implementation survey rates the adoption of OBE on a five-point scale on nine dimensions: (i) a statement of learning outcomes, (ii) communication with staff/students about the outcomes, (iii) the educational strategies adopted, (iv) the learning opportunities available, (v) the course content, (vi) student progression through the course, (vii) the assessment of students, (viii) the educational environment and (ix) student selection (Harden, 2007)
Student feedback	Anonymous collection of qualitative and quantitative student feedback for the course is crucial. To assess the sustainability attitudes in students, a questionnaire with items evaluating the concern for environmental problems; insensitivity to environmental problems; strategies for a sustainable environment; negative thinking about a sustainable environment; and importance of a sustainable environment may be designed based on published sustainability attitude scales (Richardson et al., 2016; Biasutti and Frate, 2016; Şahin and Erkal, 2017; Gupta et al., 2022; Concina and Frate, 2023; Sunthonkanokpong and Murphy, 2019; Zammitti et al., 2023)
Qualitative student and faculty feedback	Achieved through semi-structured and open-ended interviews sampled throughout the implementation of the curriculum
Formative and summative assessment of learning outcomes	In the UbD model adopted for curriculum design, formative and summative assessments indicate students’ attainment of the intended learning outcomes of the curriculum. Measures of student attainment of learning outcomes are, therefore, a means of evaluating the curriculum. Such measures include (i) the results of formative and summative assessments across the course, (ii) student engagement in research projects and faculty evaluation of student attitudes towards green and sustainable pharmacology during the projects and (iii) student authorship on papers arising from research projects (Japee and Oza, 2021)
Recognition of the curriculum	Another outcome measure will be recognition of the curriculum within the university and externally. Examples of measures will include approval of the curriculum by committees within the university, peer-reviewed publication on the curriculum, core concepts and materials developed and adoption of elements of the curriculum, core concepts and teaching materials by other universities
Qualitative and quantitative faculty surveys	Survey designed to elicit faculty feedback on curriculum design and the implementation and evaluation of the impact of education on student attitudes toward green and sustainable pharmacology
Quantitative student surveys and assessments of awareness of green and sustainable pharmacology	Quantitative Likert scale survey to evaluate both the students’ expectations and experience of the education provided and the students’ awareness of and preparedness to implement green and sustainable pharmacology. Surveys on students (a) before the first learning activity of the course and (b) within 3 weeks of the end of the course in a mixed within- and between-subjects design would provide meaningful data

## 4 Discussion

### 4.1 Drug lifecycle assessment as the source of course content

Every active synthetic drug or pharmaceutical ingredient will begin its journey as a candidate compound from a set of raw materials in a synthetic chemistry laboratory and will then be subjected to rigorous chemical modifications to enhance its pharmacological effect, reduce its toxicological effects and improve its delivery (dosage form) to the site of action. The evaluation of these candidate compounds in biological systems (cell lines, animals and eventually humans) encompasses the drug discovery and development (preclinical and clinical) phases. With regulatory oversight at all stages, the active pharmaceutical ingredient, upon clearing the clinical development stages and postmarketing surveillance phase, has found its way as a drug for veterinary or clinical use. Full-scale pharmaceutical production for use in patients then ensues. Although only a fraction of these candidate compounds ultimately become approved medicines, enormous amounts of chemicals (candidate compounds, excipients, solvents, packaging materials, etc.) and energy (for production and distribution) are consumed in every stage, along with the release of potentially toxic chemicals, carbon dioxide and nonbiodegradable materials to the environment, accounting for pharmaceutical pollution. Pharmaceutical companies package and distribute drugs for storage and use in veterinary and clinical settings. The prescribed drug (along with its excipients) is consumed and excreted by patients in a (un)metabolised form and the unconsumed drug (expired and surplus) reaches the sewerage system and is eventually released into the environment. Despite being subject to safe waste disposal systems and water recycling treatment, pharmaceuticals at lower concentrations (below the dose of observable effect) escape into the environment where they accumulate and are redistributed in the living flora and fauna (Orive et al., 2022). A critical evaluation of the integral processes involved in each stage of the drug lifecycle is necessary to incorporate green and sustainable principles to avert adverse environmental impacts.

Therefore, the course content of a green and sustainable pharmacology curriculum founded on the drug lifecycle is beneficial for identifying and addressing underlying issues. A sample list of course content topics curated on the basis of a review of the drug lifecycle (Kümmerer, 2010; Orive et al., 2022; Ritter et al., 2023; Elizalde-Velázquez et al., 2019) is presented in Supplementary Table S1. By mapping the curated content topics onto the drug lifecycle, we noted that the topics covered every stage of the drug lifecycle (Figure 3). One of the key components of course content is the development of a regulatory framework and recommendations for changes in existing legislation to achieve green practices and environmental sustainability. A recent example is the recommendations made by the PlanED Prescribing Initiative to the European Commission to strengthen environmental sustainability provisions in EU pharmaceutical legislation. Eight European medical schools, the Dutch National Institute for Public Health and the Environment, supported by others, including the European Association for Clinical Pharmacology and Therapeutics, collaborated to form the PlanED Prescribing Initiative (Piët et al., 2024). The establishment of a harmonised framework for determining and consistently reporting the environmental impact of medicines and improvements in the availability and transparency of environmental impact data were aimed at helping prescribers make environmentally sustainable choices and at requiring pharmaceutical companies to report product-level environmental-impact and sustainability data (Piët et al., 2024).

**FIGURE 3:**
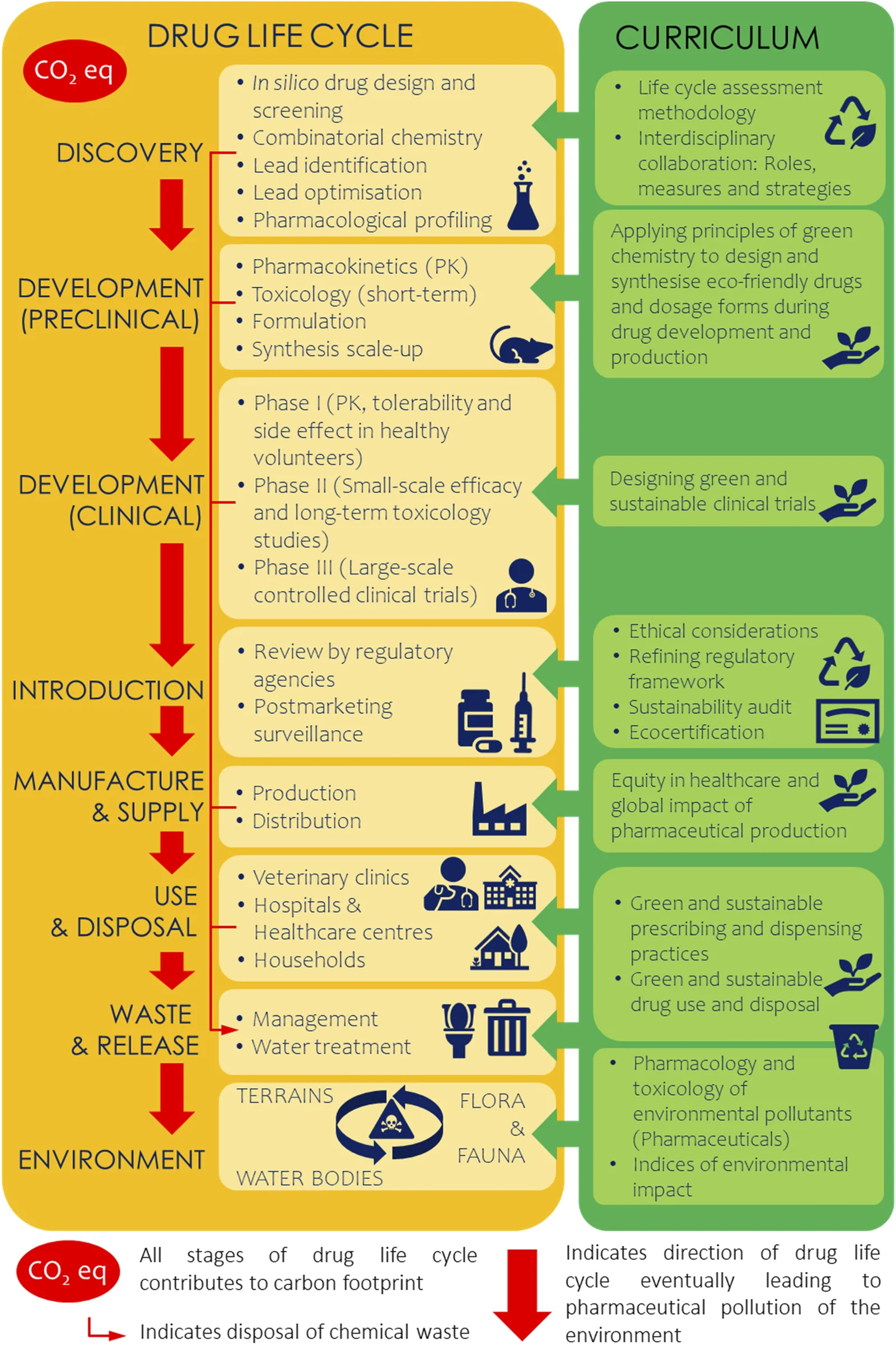
Curriculum mapping: Schematic mapping of the predicted content topics of the green and sustainable pharmacology curriculum against the drug lifecycle.

### 4.2 Core concepts

The identification of core concepts is crucial for achieving effectiveness in education, consistency in content, assessing students on “knowledge that matters” and allowing ease of comparison of the effectiveness of innovative pedagogical methods (Santiago et al., 2021; White et al., 2021; Sands et al., 2018). This demands consistency in the concepts taught and the curriculum, especially on a subject that has planetary relevance, needs to be prescriptive enough to achieve the desired learning outcomes (knowledge, skills and attributes), flexible enough to be dynamic and inclusive to achieve adaptation across the world (Wallace et al., 2021) and to permit educators and students to focus on deep learning and the development of enduring conceptual frameworks (Santiago et al., 2021). One method would be to adopt the Delphi method, as previously used to define core pharmacology concepts (Walley and Webb, 1997; Wallace et al., 2021; White et al., 2023), to distinguish inputs from national, regional and international stakeholders (e.g., pharmacology educators, industry and hospitals) on the core concepts that must be covered in a curriculum on green and sustainable pharmacology. Recently, the combination of backward design and the Delphi technique was used to develop diversity-related competencies in pharmacy education and was noted to be suitable for gaining insights into the factors needed in the educational process to achieve competencies to provide care in diverse, equitable and inclusive manners (Hardy et al., 2024). By embedding lifecycle thinking, pollution pathways and carbon/resource consequences into familiar pharmacology concepts (dose, efficacy, safety and risk–benefit), the curriculum turns green and sustainability principles into a practical competence that pharmacologists can apply immediately in research, teaching and clinical translation.

### 4.3 Programme evaluation

Programme evaluation is a comprehensive assessment of the entire educational programme that involves evaluating the mission, objective, design, implementation and outcomes of the programme, for which a systematic method to collect and analyse information is essential. Programme evaluation has a broader scope, evaluating the effectiveness of an entire educational programme, including alignment with the mission of the educational institution, and includes noncurricular aspects such as resources, administration and support aspects, whereas the curriculum evaluation described in the results section focuses specifically on the curriculum’s content, teaching methods and assessment processes.

We propose selecting qualitative and quantitative methods and stages of evaluation on the basis of published literature (Coles and Grant, 1985; Ruhe and Boudreau, 2013; Santen et al., 2019). The framework of evaluation must have specific elements such as alignment with vision, measurable outcomes, design and alignment with assessment (Pearce et al., 2015; Hays, 2016). The evaluation framework must consider factors such as how common the model is (Smidt et al., 2009; Roos et al., 2014) and the relevance of the model for the current setting (Allen et al., 2022) if the model accommodates flexibility, collaboratively engages learners in the identification of emergent needs and permits creative evolution of the programme content and process (Lieff, 2009; Frye and Hemmer, 2012). Data collection from all three interlinked parties (1) ultimate beneficiaries of the programme (by student-level assessments), (2) leaders/drivers of the curriculum (by educators and stakeholder surveys) and (3) founding/facilitating organisations (department/university-level recognition attained) is the fundamental requirement of programme evaluation. The programme evaluation of a learner-centric curriculum begins with student-level assessments. Some types of data collected from these ultimate beneficiaries of the programme include performance in course assessments, feedback on course experience, satisfaction with how the course has influenced career choice, willingness to lead research projects and publication metrics. Feedback from teaching faculty (including guest lecturers), research project supervisors, stakeholders (industry, hospitals, institutional and national regulatory agencies, etc.) and administrators will provide vital iterative inputs to the programme and indicate their influence on student-level assessments. Finally, metrics facilitating interdisciplinary efforts, recognition and publicity, agreements and partnerships with stakeholders and resource allocation by the department/university become the touchstone to monitor and encourage the programme. Table 2 presents a comprehensive framework for evidence-based programme evaluation, designed to systematically assess the impact and effectiveness of the green and sustainable pharmacology course across the student, educator and institutional levels.

**TABLE 2:** Evidence-based programme evaluation. Evidence-based programme evaluation encompassing three levels, namely, the student, educator/stakeholder and department/university levels.

Level 1 Student-level assessments	Academic performance	Performance in formative assessments (e.g., quizzes, in-class activities, reflective journals) and summative assessments (e.g., examinations, capstone projects, objective structured clinical examination) Pre- and postcourse knowledge assessments to measure learning gain attributable to the curriculum Competency mapping against defined learning outcomes (e.g., demonstration of green chemistry reasoning in drug design scenarios)
	Course experience and attitudinal shifts	Reflective assignments evaluating personal growth and shifts in environmental awareness Exit surveys capturing overall experience, satisfaction with learning gained, perceived relevance to future practice and influence of the course on career aspirations Longitudinal attitudinal surveys (e.g., at 6 and 12 months post-completion) to assess durability of mindset transformation Validated instruments measuring environmental literacy and self-efficacy in sustainable practice (Milfont and Duckitt, 2010)
	Research engagement and output	Surveys assessing interest and willingness to undertake sustainability-related research projects Quality metrics for research outputs, including conference presentations, peer-reviewed publications and citation impact Tracking of student-led sustainability initiatives or innovation projects (e.g., green drug design proposals, community outreach) Proportion of students selecting sustainability-related dissertation or elective topics
Level 2 Educator and stakeholder surveys	Teaching and delivery	Teaching faculty evaluations (self-assessment of pedagogical effectiveness, confidence in delivering green and sustainable pharmacology content and perceived curriculum coherence) Guest lecturer feedback (relevance of sessions, alignment with curriculum objectives and quality of student engagement) Research project supervisor assessments (student preparedness, quality of research questions and integration of sustainability principles) Peer observation and peer review of teaching to ensure pedagogical quality and consistency across modules Faculty development needs assessment (identifying gaps in educator training on green and sustainability topics)
	External stakeholder engagement	Feedback from industry partners, institutional and national regulatory agencies, hospitals and other healthcare settings on curriculum relevance and graduate preparedness Employer satisfaction surveys assessing the competency of graduates in sustainability-related roles Advisory board evaluations providing strategic input on curriculum alignment with evolving industry and regulatory expectations
	Operational support	Evaluation of administrative support adequacy and resource management efficiency Assessment of technological infrastructure supporting curriculum delivery (e.g., access to *in silico* tools, simulation platforms and virtual laboratories)
Level 3 Department and university-level recognitions	Interdisciplinary integration	Evidence of facilitation and institutionalisation of interdisciplinary collaborations (e.g., joint modules, cosupervised research, shared seminars) Documentation of cross-departmental governance structures supporting the curriculum (e.g., interdisciplinary curriculum committees)
	Institutional recognition and visibility	Internal and external recognition of the initiative (e.g., teaching awards, institutional commendations, media coverage) Publicity and dissemination through university communications, conferences and scholarly publications on the curriculum itself Benchmarking against peer institutions offering comparable sustainability-oriented curricula
	Graduate outcomes and career trajectories	Prediction and tracking of career success in sustainability-related roles spanning the drug lifecycle (e.g., green drug design, sustainable manufacturing, ecopharmacovigilance, regulatory affairs, sustainable prescribing) Alumni tracking surveys at defined intervals (e.g., 1, 3 and 5 years post-graduation) to assess career placement, professional impact and sustained engagement with sustainability principles Graduate destination data disaggregated by sustainability-relevant career pathways
	Partnerships and external engagement	Formal agreements and partnerships with research centres, pharmaceutical manufacturing units, regulatory agencies and hospitals Memoranda of understanding or collaborative frameworks with environmental organisations and sustainability bodies Joint grant applications or co-funded research initiatives arising from curriculum-driven partnerships
	Resource and financial sustainability	Dedicated funding allocation for curriculum development, delivery and evaluation Evidence of diversified funding streams (e.g., institutional budgets, external grants, industry sponsorship, philanthropic support) Cost-effectiveness analysis of curriculum implementation relative to measurable outcomes

### 4.4 Pedagogical strategy and curriculum iteration cycle

Teaching strategies that provide optimal utilisation of connecting new information to human memory pathways to accomplish the best possible outcomes include managing cognitive load (the degree of difficulty encountered by the learner), dual encoding (optimising visual and auditory modalities), spiral syllabus (expanding learning in a logical and sequential manner from simple base information to a comprehensive understanding of the learning outcome), bridging (the concept is reintroduced as a building block for the subsequent class) and chunking (breaking down concepts in to smaller segments for ease of understanding) (Azzam and Easteal, 2021). A constructive learning environment in which the cognitive load is optimised with a high flow experience (with a balanced challenge and skill) maximises the impact of pedagogical interventions in pharmacology (Khurshid et al., 2023). A network meta-analysis evaluating pedagogical strategies involving 21,269 students revealed that team-based learning, problem-based learning combined with case-based learning and flipped classrooms were identified as optimal strategies for pharmacology education (Xiao et al., 2023). The curriculum of green and sustainable pharmacology is proposed to begin with the fundamentals of sustainability and pharmacology, which deepen the logical sequence of the drug lifecycle (Figure 3; Supplementary Table S1), with the application of key concepts in multiple expanding layers (Harden, 1999). The concepts of green and sustainability by nature trigger affective domains related to the concerns (feelings) of negative impacts on the environment (value in preventing adversity) and empathy towards future generations (Anderson and Krathwohl, 2001). The adoption of the aforementioned teaching strategies known to promote long-term memory retention, which is necessary for student performance, in the interdisciplinary curriculum facilitates the sustainability-oriented attitudes needed to effect a change at any stage of the drug lifecycle. A participatory class environment with guest lecturers leading debates on specific topics, preclass videos, online interactions of students and educators in secure blogs, student contributions to syllabus preparation, role playing (Banos et al., 2024), gamification (Kramer et al., 2018) and case-based learning (Fasinu and Wilborn, 2024) are among the various other strategies that educators can choose from to facilitate the learning of green and sustainable pharmacology. A wide variety of interdisciplinary learning activities are available (Edelbroek et al., 2018) and one example of a participatory class environment that utilises multiple pedagogical strategies is described in Supplementary Table S2.

The curriculum iteration cycle (Figure 4) proposed for the green and sustainable pharmacology programme begins with needs analysis, the outcome of which will determine the formation of an interdisciplinary team of leaders in the institution that identifies a suitable curriculum design model (UbD and OBE in this case) and develops course content. A curriculum in which the alignment of learning outcomes, activities and assessment is achieved needs to be implemented, evaluated and integrated with the outcome of the programme evaluation to enable continuous monitoring and workplace informed value additions to the programme.

**FIGURE 4:**
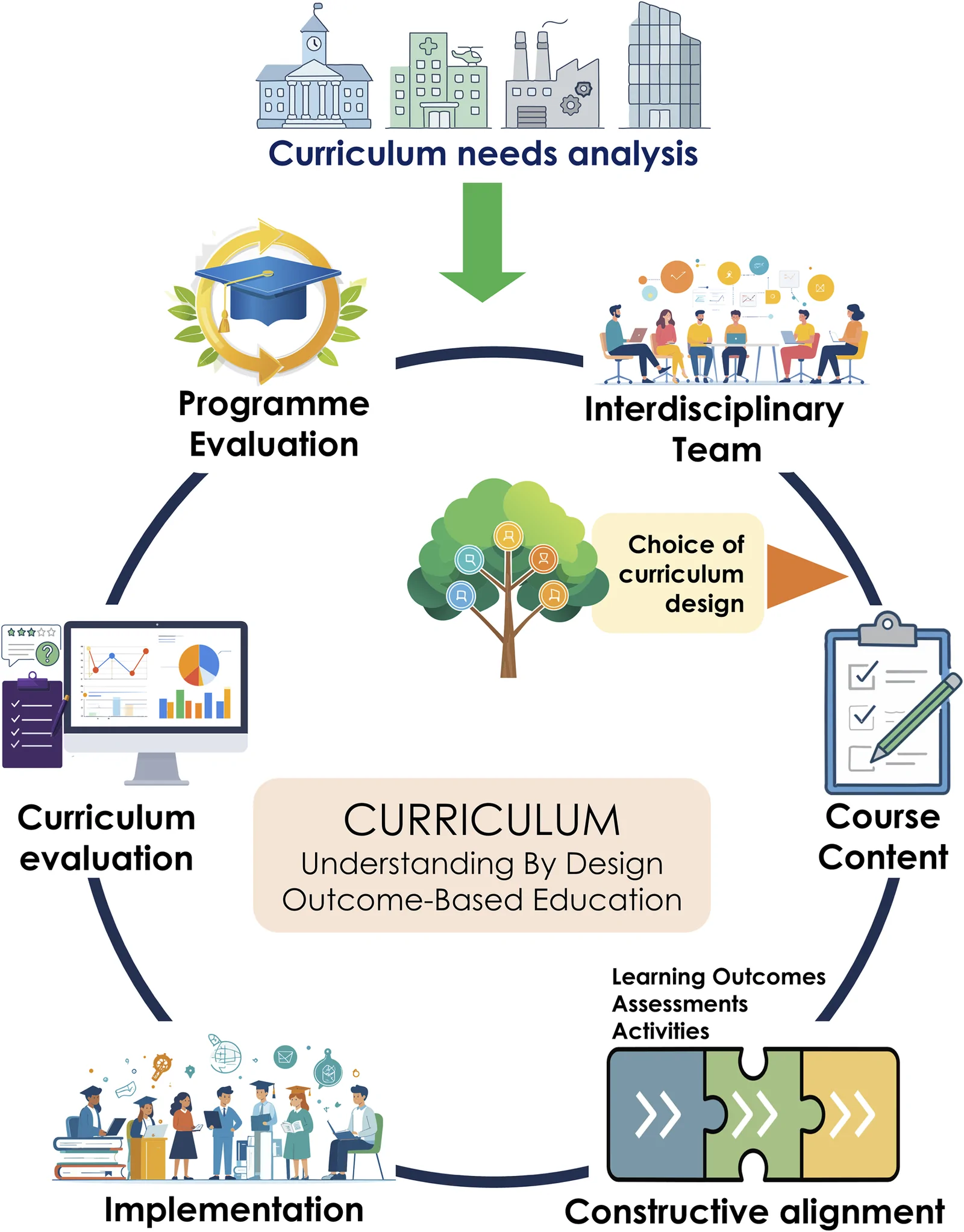
Curriculum iteration cycle: The design, implementation, and evaluation of a curriculum is a cyclic and continuous process that involves multiple stakeholders. This ensures alignment with the green and sustainability requirements of the drug lifecycle.

### 4.5 A foundation for planetary health

Planetary health is defined as “the achievement of the highest attainable standard of health, wellbeing and equity worldwide through judicious attention to the human systems (political, economic and social) that shape the future of humanity and the Earth’s natural systems that define the safe environmental limits within which humanity can flourish” (Whitmee et al., 2015). It is an emerging interdisciplinary field that synthesises established evidence to understand how environmental degradation and resource constraints shape disease risk, health system resilience and health equity, with the ultimate goal of protecting human health by safeguarding natural systems. Calls in recent years have increasingly emphasised the need to develop curricula that deliberately prepare graduates to serve as future leaders in planetary health (Brennan and Madden, 2023; Redvers et al., 2023). Several studies have begun piloting such curricula and offering practical guidance and recommendations to inform their design and implementation in medical (Slimings et al., 2022; Blom et al., 2023; Visser et al., 2024; Nash et al., 2025) and pharmacy (Urslak et al., 2025) education. Notably, the value of international and interdisciplinary frameworks in both planetary health (Park et al., 2026) and guiding natural prescriptions (Struthers et al., 2026) is well recognised. On the other hand, Bakkum et al. highlighted the challenges faced by clinical pharmacology teachers in incorporating sustainability aspects into their curriculum. These include lack of time, competing priorities, methods adopted for teaching (problem-based over traditional lectures and textbook-based methods) and the level of expertise in planetary health. The solutions proposed include (1) collaboratively creating stand points and developing teaching materials for students and teachers that are free of copyright restrictions and easy to use, revise and redistribute and (2) incorporating planetary health into pharmacotherapy education to effect changes in prescribing practices (Bakkum et al., 2022). Planetary health provides the broadest normative frame for understanding why pharmacology must reckon with its environmental footprint.

### 4.6 One Health, planetary health and green and sustainable pharmacology

Green and sustainable pharmacology and planetary health curricula share a common commitment to systems thinking, prevention-oriented practices and evidence-informed decision-making that protect human health by safeguarding natural systems. Both emphasise interconnectedness (clinical outcomes, ecosystems and equity), life-course and population impacts and the translation of knowledge into action through professional responsibility, stewardship and policy literacy. Planetary health curricula have a broader scope across the determinants of health (climate change, biodiversity loss, food and water systems, and urban design), health equity, governance and cross-sector solutions. What is unique to a green and sustainable pharmacology curriculum is its medicine-specific focus: the full drug lifecycle (discovery, manufacturing, procurement, prescribing, use and disposal), pharmaceutical pollution pathways, environmental risk assessment, ecopharmacovigilance, greener formulation and delivery choices and the carbon/resource implications of therapeutic strategies. A green and sustainable pharmacology curriculum operationalises planetary health for pharmacologists by translating broad sustainability imperatives into pharmacology-specific competencies, such as lifecycle thinking, awareness of pharmaceutical pollution pathways and the appraisal of carbon and resource implications of therapeutic choices. By embedding these dimensions alongside core pharmacological science, the curriculum strengthens the ability of pharmacologists to evaluate clinically relevant environmental trade-offs and to apply planetary health principles to research, teaching and practice without compromising patient-centred outcomes. Planetary health provides the overarching “why,” whereas green and sustainable pharmacology provides a high-impact “how” within medicines.

A recent Lancet Planetary Health Commission proposed a systems-based strategy for mitigating pharmaceutical pollution, framing it as a planetary-boundary transgression that demands coordinated action across the entire medicine life cycle (Thornber et al., 2026). The One Health framework translates planetary-scale imperatives into an operational lens for interdisciplinary action. Defined by the One Health High-Level Expert Panel (OHHLEP) as “an integrated, unifying approach that aims to sustainably balance and optimize the health of people, animals and ecosystems” (One Health High-Level Expert et al., 2022), One Health makes visible the pathways through which a prescribing decision in a clinic propagates to microbial ecologies in downstream waterways, veterinary resistance patterns, and soil microbiome integrity. The Quadripartite One Health Joint Plan of Action (2022–2026), jointly developed by WHO, FAO, WOAH, and UNEP, identifies six action tracks—including AMR, food safety, and environment and climate change, each of which intersects with pharmaceutical production, use, and disposal (World Health Organization, 2022). Despite its systems-thinking scaffold, One Health often defaults to strong anthropocentrism, weak anthropocentrism offers a more pragmatic ethical compass: it keeps patient welfare primary while recognising the intrinsic value of non-human life and ecological systems (Porat Wojakowski and Porat-Butman, 2025). This ethical reorientation, from valuing ecosystems only when they threaten human health to acknowledging their independent moral standing, provides the normative foundation upon which a green and sustainable pharmacology curriculum must be built. Green and sustainable pharmacology occupies the operational core of this nested framework, translating planetary-level imperatives and One Health interdependencies into discipline-specific competencies, metrics, and practices across the drug life cycle. Witte and Müller (2025) drew a critical distinction: sustainable pharmacy is a guiding principle that encompasses intergenerational equity, equitable access, and system-wide resource stewardship, whereas green pharmacy is a primarily ecological concept focused on reducing the environmental burden of pharmaceutical processes through green chemistry, benign-by-design drug development and waste minimisation. They identified education, environment, society, economy, pharmacology and culture as six interdependent domains that must be addressed holistically, alongside sustained dialogue on the values and ethics underpinning pharmacy and healthcare systems (Witte and Müller, 2025). At the policy level, WHO’s December 2024 call for transformative action towards greener pharmaceutical manufacturing and distribution (World Health Organization, 2024), the Lancet Planetary Health recommendations for environmentally sustainable prescribing within EU pharmaceutical legislation (Piët et al., 2024) and the International Pharmaceutical Federation’s (FIP) 2023 Statement of Policy affirming that pharmacists are “well-positioned and ethically responsible to mitigate climate and pollution risks to health throughout the pharmaceutical supply chain” (FIP, 2023) collectively signal that the profession’s regulatory and institutional architecture is converging on a mandate for sustainability literacy. Within this tripartite conceptual architecture, planetary health answers why pharmacologists must care (human flourishing depends on ecological integrity), One Health answers how impacts propagate across human, animal, and environmental compartments (providing the systems-thinking pedagogy), and green and sustainable pharmacology answers what specific knowledge, skills, and values learners must acquire to act as change agents across the medicine life cycle. This curriculum is, to the best of our knowledge, the first to explicitly integrate all three frameworks into a coherent postgraduate programme, ensuring that students do not merely learn sustainable techniques in isolation but understand them as expressions of a deeper professional and ethical commitment to planetary stewardship.

### 4.7 Justice, equity and access-to-medicines trade-offs

The pursuit of green and sustainable pharmacology raises justice and equity questions that are inseparable from its environmental objectives. Pharmaceutical pollution and environmental degradation do not fall evenly. Poor or marginalised communities who already have reduced access to the healthcare and economic benefits of pharmaceuticals are often disproportionately exposed to the environmental harms of pharmaceutical contamination, particularly in low- and middle-income countries (LMICs) where pollution levels are reportedly higher (Thornber et al., 2026). Conversely, environmentally preferable manufacturing pathways or greener formulations may carry higher costs, risking further constraints on equitable access, a tension the WHO has explicitly acknowledged, stating that greener pharmaceuticals must not come at the expense of access, quality, or safety (World Health Organization, 2026). Sustainable production of medicines is a broad undertaking that must simultaneously encompass equitable access to healthcare, ethical research and production practices and environmental stewardship (Noonan et al., 2024). These trade-offs are not merely logistical but fundamentally ethical and political, underscoring the need for curricula in green and sustainable pharmacology to equip learners with the capacity to reason through justice-informed dilemmas that balance ecological imperatives against the right to affordable, accessible medicines, particularly for the most vulnerable populations. A recent report demonstrated that commonly used bioethics theories including deontology, consequentialism, contractualism, modern casuistry, justice theories and feminist approaches, each offer distinct and complementary lenses for navigating the moral tensions that arise in efforts to move towards sustainable healthcare. This casuistic approach, for example, illustrates how clinicians can reason through inhaler-switching decisions case by case, respecting critical distinctions between patients who can and cannot use alternative devices (Valentina and Charles, 2025). Embedding such structured ethical deliberation within our curriculum gives learners a principled methodology for weighing ecological imperatives against the right to accessible, affordable medicines, particularly for the most vulnerable populations.

### 4.8 Training learners to navigate trade-offs between environmental and patient-centred outcomes

#### 4.8.1 Structured decision frameworks

Emerging methodological guidance demonstrates that clinical and environmental goals can be systematically co-evaluated rather than treated as competing priorities. A stepwise approach for integrating environmental sustainability into evidence-based clinical guidelines through the GRADE Evidence-to-Decision framework, provides learners with a replicable methodology for weighing environmental impact alongside clinical effectiveness and patient values (van Nieuwenhuizen et al., 2025). This is complemented by the Sustainability in Quality Improvement (SusQI) framework, which operationalises trade-off navigation by measuring the sustainable value of healthcare interventions (defined as health outcomes assessed against environmental, social, and economic costs) and embeds these metrics directly into established clinical quality improvement cycles (Stanford et al., 2025; Chambers et al., 2024). Through SusQI-based projects, learners acquire a structured, repeatable method for evaluating trade-offs rather than relying on *ad hoc* intuition.

#### 4.8.2 Case-based dilemma training

Our curriculum incorporates structured clinical scenarios in which learners must weigh therapeutic equivalence against environmental metrics (including carbon footprint, ecotoxicity, and pharmaceutical waste) under realistic constraints of cost and patient access. This pedagogical approach aligns with recommendations that sustainability training should favour systemic thinking, critical analysis, and ethical decision-making through interdisciplinary, case-based learning integrated within clinical coursework (Almeida, 2026; Kennie-Kaulbach et al., 2024). Through iterative exposure to such dilemmas, learners develop the deliberative reasoning skills necessary to identify when environmental gains are achievable without clinical compromise, and when patient-centred outcomes must take precedence.

#### 4.8.3 Real-world prescribing evidence

The most compelling pedagogical tool is concrete clinical evidence demonstrating that the perceived trade-off is frequently a false dilemma. For example, switching patients from pressurised metered-dose inhalers (pMDIs) to dry powder inhalers (DPIs) maintained disease control in both asthma and COPD while achieving a 20–40-fold reduction in carbon footprint (Janson et al., 2025) and combination DPIs reduce the carbon footprint of asthma care by approximately 95% while simultaneously improving disease control (Wilkinson and Woodcock, 2024). Similarly, evidence from deprescribing interventions demonstrates that reducing unnecessary polypharmacy simultaneously decreases pharmaceutical waste and improves patient safety by lowering adverse drug events and hospitalisation rates (Japelj et al., 2024). These real-world cases transform the abstract principle into teachable, evidence-based practice, enabling learners to recognise that environmentally sustainable prescribing and optimal patient outcomes are more often synergistic than antagonistic.

### 4.9 Implementation challenges and plausible solutions

The implementation of a new curriculum is challenging for both fully mature and maturing educational institutions. Faculty members and students may resist changes because of comfort with the existing curriculum or scepticism about the benefits of the new curriculum. This may be addressed by revealing the outcome of needs analysis and assuring faculty members about the purpose and benefits of the course, its alignment with the vision of the institution and how it will meet the demands of the workplace on the student. Training, exposure to the green and sustainability landscape through (inter)national conferences, interdisciplinary partnerships with faculty of other relevant departments and other professional development initiatives need to be provided to faculty to ensure that they are comfortable and competent in delivering the new content. The development and implementation of a new curriculum requires additional resources, including time, personnel and financial investment, which can be met by carefully conducting a needs analysis to identify resource gaps and by seeking additional funding from granting agencies or partnerships with industries and hospitals to support implementation. Leveraging technology and online resources helps reduce costs and enhance learning opportunities. Ensuring that the new curriculum aligns with accreditation standards and integrates smoothly with existing courses is a complex task. The interdisciplinary team will involve curriculum experts and accrediting bodies in the planning process to ensure alignment with standards. The development of new assessment tools that align with the objectives of the new curriculum may be perceived as challenging. In addition to the UbD model, designing assessments that are competency-based and focus on real-world skills and implementing the continuous feedback and evaluation mechanisms described in the programme evaluation (Figure 4) are expected to address this challenge. The green and sustainable pharmacology curriculum relies on how existing expertise and resources can be restructured to meet the requirements of needs analysis. An interdisciplinary curriculum is built on the collegial collaboration of experts from diverse disciplines to address an emerging concern with planetary significance. Students nurtured in this curriculum are more likely to use a value-based approach when they function in the interdisciplinary workforce.

#### 4.9.1 Tiered implementation pathway

While the proposed green and sustainable pharmacology curriculum offers a comprehensive vision grounded in Understanding by Design (UbD) and Outcome-Based Education (OBE), its translation into practice demands a pragmatic, staged implementation pathway (Figure 5) that accommodates the considerable heterogeneity in institutional capacity worldwide. We therefore propose a tiered model comprising three progressive levels. Tier 1 (Minimal Viable) constitutes a low-barrier entry point requiring neither new courses nor additional personnel; modelled on the University of Huddersfield approach, a single faculty champion reframes 4–6 h of existing pharmacology content through an environmental lens, incorporating landmark cases such as diclofenac-induced vulture decline, a drug lifecycle overview and responsible disposal practices, supplemented by targeted assessment items and a pre/post awareness survey, all achievable within one semester. Tier 2 (Intermediate) activates the curriculum’s constructive alignment framework and spiral syllabus strategy through a dedicated 15–20 h module developed by an interdisciplinary team of 3–4 faculty, with formal learning outcomes, problem-based and case-based pedagogies, structured evaluation using validated sustainability attitude scales and at least one external partnership, typically requiring 1–2 academic years to pilot. Tier 3 (Advanced) realises the full curricular vision: a 30–40 h standalone programme encompassing the complete drug lifecycle, experiential placements, interdisciplinary research projects, capstone assessments, longitudinal attitudinal tracking and the complete programme evaluation framework deployed across student, educator and institutional levels, steered by a dedicated task force with external advisory engagement over 2–3 academic years. Critically, all three tiers share the same foundational design logic, backward design, drug lifecycle mapping, OBE-aligned assessment and an iterative cycle of implementation, evaluation and refinement, ensuring pedagogical coherence regardless of scale. The strength of a tiered pathway lies not in lowering ambition but in dismantling the inertia of perceived complexity, because a curriculum that no institution can feasibly adopt is, in practice, no curriculum at all.

**FIGURE 5:**
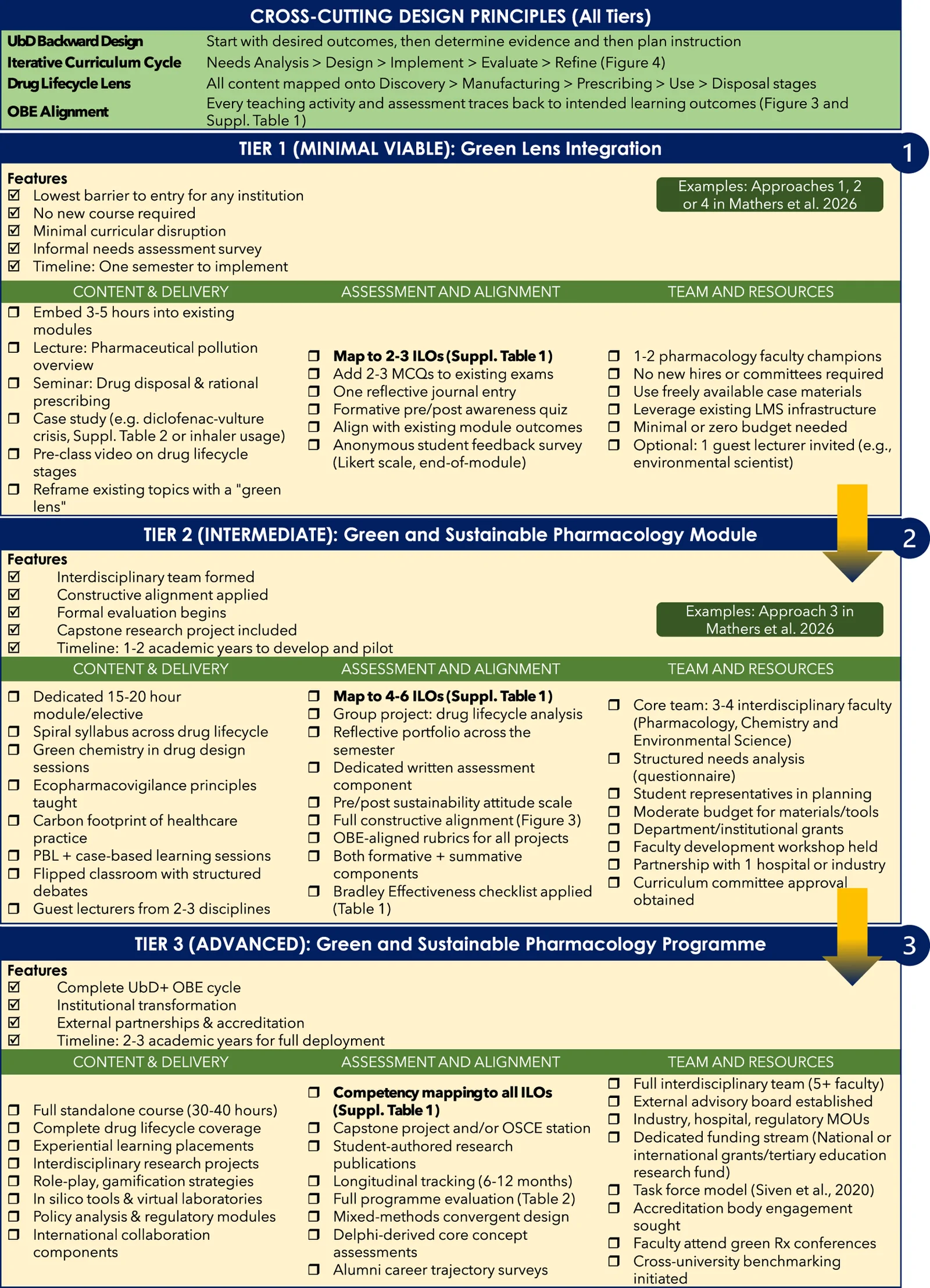
Tiered implementation pathway. Concise implementation model that distinguishes between minimal, intermediate and advanced versions of the curriculum.

## 5 Limitations of the white paper and recommendations for further study

Several limitations of this white paper warrant acknowledgement. While the curriculum design draws upon established pedagogical models notably, Understanding by Design and Outcome-Based Education and is informed by published evidence from institutions such as the Universities of Huddersfield and Helsinki, the specific configuration of content topics, constructive alignment, assessment strategies and programme evaluation metrics presented here has not been subjected to a formal pilot study. Consequently, the anticipated learning outcomes, attitudinal shifts and competency gains remain theoretical projections rather than empirically validated endpoints. Until prospective implementation data are collected across diverse institutional contexts, the transferability and effectiveness of the proposed framework cannot be confirmed.

The white paper assumes a baseline level of institutional infrastructure, faculty expertise and administrative support that may not be uniformly available across educational settings worldwide. Resource constraints including limited access to interdisciplinary collaborators, restricted funding for curriculum development, competing demands on already saturated timetables and varying degrees of institutional autonomy in curriculum reform may impede adoption. This may be expected particularly in low- and middle-income settings where the burden of pharmaceutical pollution is often most acute. Although the tiered implementation pathway is designed to mitigate such barriers, even the minimal viable tier presupposes faculty willingness and protected time for curriculum modification.

Finally, the environmental impact data underpinning several proposed content topics remain incomplete for a substantial proportion of marketed medicines. Comprehensive lifecycle assessments, carbon footprint analyses and ecotoxicological profiles are available for only a limited subset of pharmaceuticals, and standardised, transparent reporting of product-level environmental data by the pharmaceutical industry is still in its infancy. This evidence gap may constrain the depth and specificity with which certain learning activities and case studies can be developed, necessitating iterative content updates as the evidence base matures.

Several avenues of investigation are essential to advance the framework proposed in this white paper. First, prospective pilot studies implementing the tiered curriculum across institutions with varying resource levels are needed to generate empirical evidence on feasibility, learning gains and attitudinal shifts. Second, a Delphi study engaging international stakeholders, namely, pharmacology educators, industry representatives, regulatory professionals and healthcare practitioners should be conducted to validate and refine the proposed core concepts for green and sustainable pharmacology. Third, longitudinal cohort studies tracking graduates’ professional behaviour, prescribing practices and sustainability-related career trajectories would provide critical evidence on whether curricular exposure translates into sustained workplace impact. Fourth, comparative effectiveness research evaluating different pedagogical strategies (problem-based learning, gamification, experiential placements) within the curriculum is warranted to identify optimal approaches for different learner populations. Finally, collaborative efforts to develop openly accessible, standardised teaching materials, including case studies anchored to emerging environmental impact data, would facilitate global adoption and enable meaningful cross-institutional comparison of curricular outcomes.

## 6 Conclusion

The drug lifecycle poses a profound and escalating threat to environmental health. There is an urgent need to address pharmaceutical pollution and embed sustainable healthcare strategies that go beyond merely reducing carbon footprints and plastic waste (Thornber et al., 2022). Achieving meaningful upstream control and surveillance of these emerging environmental challenges demands more than policy reform or technological innovation alone. A fundamental shift in how professionals who navigate the drug lifecycle are trained to think and act is needed. The urgent need for enhanced dialog between pharmaceutical R&D and environmental science communities (Vidaurre et al., 2024) further underscores the necessity of a curriculum that equips pharmacologists with interdisciplinary competencies to bridge drug design and environmental stewardship.

Pharmacology professionals, uniquely positioned at the nexus of basic sciences and clinical medicine, represent the most strategic cohort to be equipped with these competencies. A purpose-built green and sustainable pharmacology curriculum will cultivate a new generation of professionals capable of developing, driving and operationalising holistic sustainability frameworks across the pharmaceutical and healthcare sectors (Van Wilder et al., 2024). Such a curriculum seeks to move learners from concerned global citizens who merely acknowledge sustainability challenges towards self-directed pharmacologists empowered with personal agency to effect tangible change across the workplace and beyond.

Recent systematic (Bray et al., 2023) and scoping (Visser et al., 2024) reviews have identified two critical gaps: the absence of a curriculum that meaningfully integrates green and sustainability principles into healthcare education and a paucity of literature on methodologies for developing such a holistic curriculum. This white paper addresses both gaps by proposing a comprehensive cyclic model of curriculum design, development and evaluation, grounded in a carefully curated recipe of elements (Figure 4) that collectively guide the creation and continuous refinement of the green and sustainable pharmacology curriculum. The model synergises Understanding by Design and Outcome-Based Education frameworks, from curriculum needs analysis to programme evaluation. We envision the green and sustainable pharmacology curriculum, woven into the fabric of undergraduate and postgraduate medical education, as a transformative strategy to nurture a new generation of eco-conscious pharmacologists who do not merely participate in the drug lifecycle but lead its reimagination because the future of healthcare cannot be truly healthy if the planet it serves is not.
