# Supplementary material for: Teaching the true cost of medicines: white paper for a green and sustainable pharmacology curriculum
Source: Front Pharmacol. 2026 Sep 30;17:1912700. doi: 10.3389/fphar.2026.1912700 (PMC13662423; doi:10.3389/fphar.2026.1912700)
Supplement: Supplementary file 1 [file Supplementaryfile1.pdf]

Supplementary material

Supplementary table 1: Proposed course content and learning outcomes.

**Proposed course content (CC) and intended learning outcomes (ILOs)**
CC1: Principles of sustainability, green processes and ecopharmacovigilance<br>ILO: Explain new terminologies prevalent in this interdisciplinary field
CC2: Why should pharmacologists and healthcare professionals care about the environmental consequences of medicines e<br>ILO: Recognise why change is necessary and feel professionally compelled to enact it.
CC3: Pharmacology and toxicology of environmental pollutants<br>ILOs: Explain new terminologies prevalent in this interdisciplinary field<br>Catalogue pharmaceutical pollutants<br>Estimate adverse effects of pharmaceutical pollutants based on the mechanisms of action
CC4: Life cycle assessment methodology and indices of environmental impact<br>ILOs: Evaluate the use of the life cycle assessment methodology to critically examine the impact of various stages of drug development on the environment
CC5a: Applying principles of green chemistry to design and synthesis of eco-friendly drugs and dosage forms during drug development and production<br>ILOs: Investigate sustainable drug discovery and development approaches, including green synthesis techniques, renewable feedstocks, and reduced energy consumption<br>Integrate green and sustainability criteria, such as eco-toxicity, biodegradability, and carbon footprint, into the evaluation and selection of pharmaceuticals and related materials such as those used as excipients and in packaging
CC5b: Designing green and sustainable clinical trials<br>ILO: Integrate green and sustainability criteria, such as ecotoxicity, biodegradability, and carbon footprint to stages of clinical trials
CC5c: Green and sustainable prescribing and dispensing practices<br>ILO: Predict strategies for minimising pharmaceutical waste generation, promoting recycling and reuse, and implementing proper disposal methods to mitigate environmental pollution
CC5d: Green and sustainable drug use and disposal<br>ILO: Examine the current analytical methods of estimation of pharmaceuticals in the environmental samples and monitoring systems developed to estimate release of pharmaceutical waste<br>Predict strategies for minimising pharmaceutical waste generation, promoting recycling and reuse, and implementing proper disposal methods to mitigate environmental pollution
CC6: Ethical considerations, regulatory framework and guidelines, sustainability audit, and certification<br>ILO: Describe regulatory frameworks and ethical considerations, guidelines related to environmental sustainability in pharmaceutical manufacturing and healthcare practices, including green labelling and eco-certification.
CC7: Equity in healthcare and the global impact of pharmaceutical production<br>ILO: Develop strategies to achieve sustainable access to quality health care services, need-based distribution of resources and minimise disparities in providing healthcare
CC8: Interdisciplinary collaboration: Roles, measures and strategies<br>ILO: Develop skills to effectively communicate the importance of green and sustainable pharmacology principles to stakeholders, advocate for environmentally responsible practices, and promote sustainable initiatives within the pharmaceutical industry and healthcare sector
CC9: Problem-based learning using case studies and success stories<br>ILO: Analyse case studies and appreciate the success stories in green and sustainable processes that inspire its application pharmaceutical industry

Supplementary table 2: An example lesson plan that incorporates multiple pedagogical strategies to achieve participatory class environment .

Title	Lessons from a catastrophic effect of a pharmaceutical pollution on the environment
**Intended Learning outcomes**	Estimate adverse effects of pharmaceutical pollutants based on the mechanisms of action.<br>Collaboratively evaluate the use of the life cycle assessment methodology to critically examine the impact of various stages of drug development on the environment.<br>Integrate the principles of green and sustainability to solve a case study on the adverse effect of a pharmaceutical on the environment<br>Predict strategies for minimising pharmaceutical waste generation, promoting recycling and reuse, and implementing proper disposal methods to mitigate environmental pollution<br>Examine the current analytical methods of estimation of pharmaceuticals in the environmental samples and monitoring systems developed to estimate release of pharmaceutical waste<br>Describe regulatory frameworks and ethical considerations, guidelines related to environmental sustainability in pharmaceutical manufacturing and healthcare practices, including green labelling and eco-certification.<br>Analyse case studies and appreciate the success stories in green and sustainable processes that inspire its application pharmaceutical industry.
**Pre-class material**	Video clip and a couple of scientific publications on the effect of diclofenac on vulture population.
**Pre-class activity**	Students will be asked to post their opinions/initial thoughts on the case on an instructor-controlled online platform
**Pre-class planning**	Students will be grouped to play the roles including but not limited to (a) Animal activists, (b) Industrial experts, (c) Regulatory agencies, (d) Environmental toxicologists. They will approach the case with the above perspectives and identify strategies to minimise, monitor and regulate the release pharmaceuticals into the environment. The student groups will collate the evidence and key points for discussion from each of the above perspective and bring it to class for the debate.
**In-class activity**	1. The groups will be given preparatory time to organise their discussion points generated during the pre-class planning<br>2. Representatives from each group will place their argument and propose strategies that may be supported or rejected by members of other group with justifications.<br>3. The entire session will be moderated by a panel of lecturers and guest experts who will provide appropriate feedback on the debate.
**Post-class activity**	Each student will write a reflective report on the entire case taking into consideration all stages of the drug lifecycle and strategies to minimise the adverse impact on the environment.<br>They will also critique on the initial comments posted during the pre-class activity based on the collaborative knowledge acquired during the debate.
**Assessment**	Assessment rubrics with emphasis to elements namely, depth of the content, application of green and sustainability principles and integrating it with pharmacology knowledge, presentation skills, justification of arguments, openness to accept criticism, and evidence of systems thinking
